# Supplementary material for: Smart and low-cost fluorometer for identifying breast cancer malignancy based on lipid droplets accumulation
Source: PLoS One. 2023 Dec 21;18(12):e0294988. doi: 10.1371/journal.pone.0294988 (PMC10735024; doi:10.1371/journal.pone.0294988)
Supplement: S1 Fig — (DOCX) [file pone.0294988.s001.docx]

Look in the chart’s data table for more information.

A B C D E F G H R I S J T U V W K L
