# Supplementary material for: Smart and low-cost fluorometer for identifying breast cancer malignancy based on lipid droplets accumulation
Source: PLoS One. 2023 Dec 21;18(12):e0294988. doi: 10.1371/journal.pone.0294988 (PMC10735024; doi:10.1371/journal.pone.0294988)
Supplement: Attachment — Submitted filename: Response to Reviewers.docx [file pone.0294988.s002.docx]

1. Is the manuscript technically sound, and do the data support the conclusions?

The manuscript must describe a technically sound piece of scientific research with data that supports the conclusions. Experiments must have been conducted rigorously, with appropriate controls, replication, and sample sizes. The conclusions must be drawn appropriately based on the data presented.

Reviewer #1: Partly

Reviewer #2: Yes

2. Has the statistical analysis been performed appropriately and rigorously?

Reviewer #1: No

Reviewer #2: Yes

3. Have the authors made all data underlying the findings in their manuscript fully available?

The [PLOS Data policy](https://can01.safelinks.protection.outlook.com/?url=http%3A%2F%2Fwww.plosone.org%2Fstatic%2Fpolicies.action%23sharing&data=05%7C01%7Caditya.mandapati%40usask.ca%7C948efdbc8a2a45c1e48508db9f70708b%7C24ab6cd0487e47229bc3da9c4232776c%7C0%7C0%7C638279078779030819%7CUnknown%7CTWFpbGZsb3d8eyJWIjoiMC4wLjAwMDAiLCJQIjoiV2luMzIiLCJBTiI6Ik1haWwiLCJXVCI6Mn0%3D%7C3000%7C%7C%7C&sdata=foRafa6uVU4xljnPewDWxlcLTkmiFh6GvwPlN%2B4Aq6c%3D&reserved=0) requires authors to make all data underlying the findings described in their manuscript fully available without restriction, with rare exception (please refer to the Data Availability Statement in the manuscript PDF file). The data should be provided as part of the manuscript or its supporting information, or deposited to a public repository. For example, in addition to summary statistics, the data points behind means, medians and variance measures should be available. If there are restrictions on publicly sharing data—e.g. participant privacy or use of data from a third party—those must be specified.

Reviewer #1: No

Reviewer #2: Yes

4. Is the manuscript presented in an intelligible fashion and written in standard English?

PLOS ONE does not copyedit accepted manuscripts, so the language in submitted articles must be clear, correct, and unambiguous. Any typographical or grammatical errors should be corrected at revision, so please note any specific errors here.

Reviewer #1: Yes

Reviewer #2: Yes

5. Review Comments to the Author

Please use the space provided to explain your answers to the questions above. You may also include additional comments for the author, including concerns about dual publication, research ethics, or publication ethics. (Please upload your review as an attachment if it exceeds 20,000 characters)

Review_Manuscript Number: PONE-D-23-02819

**Reviewer #1:** 
The work presented through “Smart and low-cost fluorometer for identifying breast cancer malignancy based on lipid droplets accumulation” is highly interesting with overall quality photographs and compact presentation.

**Reviewer Comment:** The claim “The goal of applying BODIPY probe is to target specific biological pathways at the cellular and molecular level. This means that the probes do not emit fluorescence unless they are fluorescently switched on. In other words, the dye is activated by specific cellular conditions or the presence of overexpressed receptors in cancer microenvironment.” is very important for the present work and should be justified in the text either through proper references or through further experiments.

**Answer:** We agree with this feedback. The paragraph has been reworded to reflect the use of BODIPY as a fluorescent dye that was used due to its binding to neutral lipid droplets in cells. Changes made to page 4, line 4 onwards.

**Reviewer comment:** Does BODIPY stain ordinary oil droplets also? If so, the efficacy of the fluorometer should be tested upon ordinary oil droplets as positive control.

**Answer:** Yes, BODIPY can stain oil droplets and has been used as such previously. However, as we are using a cell-based system, the proposed use of ordinary oil droplets as a positive control may not be appropriate.

**Reviewer comment:** Authors should elaborate the difference in the level of lipid droplets in normal breast cells and cancerous breast cells.

**Answer:** The differences between MCF10A cells (normal breast epithelium cells) vs breast cancer cells (MCF7 and MDA-MB-231) have been elaborated on more specifically on page 2, line 25 onwards.

**Reviewer comment:** As a negative control, there should be a check on non-cancerous breast cells MCF10A to get a complete picture of the efficacy of the developed fluorometer.

**Answer:** We agree with this feedback and have therefore included BODIPY-stained MCF10A samples as part of the revised manuscript.

**Reviewer comment:** Authors should also elaborate the proposed use of the developed device in real-life conditions, i.e., starting from the biopsy to the detection. What to detect? How to detect? How it is advantageous over the currently existing practices should be properly explained in the manuscript. For this, there should be a separate section along with a graphical presentation.

**Answer:** The potential applications of our proposed fluorometer extend prominently to the domains of early detection and screening. This include complementing the advancements in wireless capsule endoscopy that introduces a new dimension of assessing tissue characteristics by considering the lipid droplets accumulation.

In the context of early detection and screening, our fluorometer's ability to detect cancer malignancy through lipid droplet accumulations holds promise for providing a non-invasive and cost-effective method for identifying cancerous cells at an early stage. This can play a crucial role in identifying potential cases for further accurate evaluations.

**Reviewer comment:** The data should be statistically verified and presented accordingly.

**Answer:** The statistical evaluation including the calculation of sensitivity, accuracy, specificity and precision have been elaborated on more specifically on page 7. Fig. 9 also demonstrates the confusion matrix drawn from the measurements obtained with our prototype.

**Reviewer #2:**

In this manuscript, the authors introduce a new fluorometer operating on fluorescence spectroscopy principles. The primary objective of their device is the identification of lipid droplet accumulation within fluorescently labeled breast cancer cells. The optimization of both the light source and optical sensor has been undertaken to ensure the proper functionality of this apparatus. By detecting fluctuations in lipid droplet levels, the fluorometer exhibits the capability to discern between moderately malignant and highly malignant cancer cell lines. Before arriving at a decision regarding publication, the reviewer wants to raise a few comments/issues:

**Reviewer Comment:** While the proposed device offers substantial merit, its sensitivity appears comparatively low in relation to other existing devices. It would be valuable for the authors to elucidate scenarios in which this lower sensitivity remains acceptable or even advantageous.

**Answer:** The reviewer's observation regarding the sensitivity of our proposed device compared to existing technologies is acknowledged. It's important to understand that, despite the fact that our device's sensitivity may seem comparatively lower, it should be assessed in light of both the intended use and the larger clinical landscape. Our device's affordability and portability make it suitable for deployment in regions with limited resources. In such settings, even a device with slightly lower sensitivity can play a crucial role in identifying potential cases for further accurate evaluations, thus contributing to early intervention. Moreover, in cases where frequent monitoring is required, such as post-treatment follow-up, a less sensitive device may still provide valuable insights. It can help in tracking changes over time and alerting healthcare providers to any deviations from the baseline.

**Reviewer Comment:** The authors present the spectral response under various wavelengths for BODIPY-stained MCF7 and MDA-MB-231 cells. To bolster their argument, it would be prudent to also provide the spectral response under different wavelengths (instead of only one wavelength) for BODIPY unstained MCF7 and MDA-MB-231, enhancing the strength of their conclusions.

**Answer:** We have studied the emitted fluorescence signal under four different LED wavelengths to compare the resulting signals from MDA-MB-231 slides, MCF7 slides and MCF10A in section 3. The average fluorescence intensity of 52 slides captured by the spectral detector under varying excitation wavelengths is observed in Fig. 5.

**Reviewer Comment:** Fig. 5 and Fig. 6 appear to contain identical data and information. To avoid redundancy, it is recommended that the authors eliminate one of these figures.

**Answer:** We agree with this feedback. We have eliminated figure 6.

**Reviewer Comment:** To enhance visual clarity, the authors should consider employing distinct colors to differentiate between the two curves presented in Fig. 2.

**Answer:** We agree with this feedback and have carefully considered your suggestions and made the necessary revisions to Fig. 2.

6. PLOS authors have the option to publish the peer review history of their article ([what does this mean?](https://can01.safelinks.protection.outlook.com/?url=https%3A%2F%2Fjournals.plos.org%2Fplosone%2Fs%2Feditorial-and-peer-review-process%23loc-peer-review-history&data=05%7C01%7Caditya.mandapati%40usask.ca%7C948efdbc8a2a45c1e48508db9f70708b%7C24ab6cd0487e47229bc3da9c4232776c%7C0%7C0%7C638279078779030819%7CUnknown%7CTWFpbGZsb3d8eyJWIjoiMC4wLjAwMDAiLCJQIjoiV2luMzIiLCJBTiI6Ik1haWwiLCJXVCI6Mn0%3D%7C3000%7C%7C%7C&sdata=95rKNFIsZGoOVgARWMOLmbeZRa4G7djCBgZt76%2FE05Q%3D&reserved=0)). If published, this will include your full peer review and any attached files.


If you choose “no”, your identity will remain anonymous but your review may still be made public.


**Do you want your identity to be public for this peer review?** For information about this choice, including consent withdrawal, please see our [Privacy Policy](https://can01.safelinks.protection.outlook.com/?url=https%3A%2F%2Fwww.plos.org%2Fprivacy-policy&data=05%7C01%7Caditya.mandapati%40usask.ca%7C948efdbc8a2a45c1e48508db9f70708b%7C24ab6cd0487e47229bc3da9c4232776c%7C0%7C0%7C638279078779030819%7CUnknown%7CTWFpbGZsb3d8eyJWIjoiMC4wLjAwMDAiLCJQIjoiV2luMzIiLCJBTiI6Ik1haWwiLCJXVCI6Mn0%3D%7C3000%7C%7C%7C&sdata=eSAaEM%2FGOFbZxs%2BQJpPsdHXDq1J7x04sSaiO1n7szg0%3D&reserved=0).

Reviewer #1: No

Reviewer #2: No
